# Proceedings of the Dental College Association

**Published:** 1863-04

**Authors:** J. Taft


### Proceedings of Societies.

### PROCEEDINGS OF THE OHIO DENTAL COLLEGE ASSOCIATION.

The Association met at the lecture room of the Ohio Dental College, February 20th, 1863.

Dr. Bonsall in the Chair.

There were present, Drs. Bonsall, Taylor, Collins, H. A. Smith, H. R. Smith, Driggs, De Camp, and Taft.

Minutes of the last meeting were read.

The Treasurer presented his report, which is as follows:

On motion, Dr. S. Driggs, of Lexington, Ky., was elected a member of the Association.

Dr. Taylor made some remarks explanatory of the financial condition of the Institution, tending to show that some effort was necessary on the part of the members of the Association to relieve the embarrassment.

Letters were read from various stock-holders of a very encouraging character.

On motion of Dr. Taft,

*Resolved*, That we proceed at the earliest practicable period to ascertain how much stock can be obtained of the members of this Association and others for the purpose of relieving the Institution of its present financial embarrassment.

*Resolved*, That the following named persons be appointed a committee to carry out the above resolution, viz.:

James Taylor, J. Taft, W. W. Allport, E. Peebles, E. Collins, S. Driggs, M. De Camp, J. M. Brown, Joseph Richardson.

Dr. Taylor read the following communication in reference to the injunction against the Treasurer of Hamilton County, restraining him from collecting the tax upon the College property:

DR. JAMES TAYLOR:—

*Dear Sir:*—

The case of the Ohio College of Dental Surgery has been heard before the Superior Court of Cincinnati, and decreed in favor of the Trustees and against the Treasurer of Hamilton County, and is now awaiting the action of the Attorney for the County, who says he will move for a new trial. The prospect is that the case will be carried up to the Supreme Court, but I have no fear of the final result in our favor.

WILLIAM FERGUSON,
*Attorney.*

The Treasurer reported the following, viz.:

The interest on the mortgages has been paid up to the first of July next, and the insurance to September next; and further, that all necessary repairs on the building have been made.

*Resolved*, That we recommend to the Board of Trustees the establishment of a Chair of Adjunct Professor of Mechanical and Operative Dentistry, this to be substituted for the demonstratorship of these departments.

*Resolved*, That Dr. E. Collins, of Connersville, Indiana, be nominated to the Trustees to fill the above Chair.

The resignation of Dr. C. B. Chapman, as Professor of Chemistry, was received, to be presented to the Board of Trustees.

The transfer of Dr. H. A. Smith from the Chair of Anatomy to that of Chemistry was recommended.

Dr. Geo. E. Jones was nominated to fill the Chair of Anatamy.

The following persons were elected officers of the Association for the ensuing year:

President, Dr. Charles Bonsall; 1st Vice President, Dr. Geo. W. Keely; 2d Vice President, Dr. Geo. Watt; Secretary, Dr. J. Taft; Treasurer, Dr. James Taylor.

Adjourned to meet on Wednesday preceding the 20th of February, 1864.

J. TAFT,
*Secretary.*

